# The Costs of Delivering Integrated HIV and Sexual Reproductive Health Services in Limited Resource Settings

**DOI:** 10.1371/journal.pone.0124476

**Published:** 2015-05-01

**Authors:** Carol Dayo Obure, Sedona Sweeney, Vanessa Darsamo, Christine Michaels-Igbokwe, Lorna Guinness, Fern Terris-Prestholt, Esther Muketo, Zelda Nhlabatsi, Integra Initiative, Charlotte E. Warren, Susannah Mayhew, Charlotte Watts, Anna Vassall

**Affiliations:** 1 Department of Global Health and Development, London School of Hygiene and Tropical Medicine, London, United Kingdom; 2 Population Council, Nairobi, Kenya; 3 Family Health Options Kenya, Nairobi, Kenya; 4 Family Life Association of Swaziland, Manzini, Swaziland; Groningen Research Institute of Pharmacy, NETHERLANDS

## Abstract

**Objective:**

To present evidence on the total costs and unit costs of delivering six integrated sexual reproductive health and HIV services in a high and medium HIV prevalence setting, in order to support policy makers and planners scaling up these essential services.

**Design:**

A retrospective facility based costing study conducted in 40 non-government organization and public health facilities in Kenya and Swaziland.

**Methods:**

Economic and financial costs were collected retrospectively for the year 2010/11, from each study site with an aim to estimate the cost per visit of six integrated HIV and SRH services. A full cost analysis using a combination of bottom-up and step-down costing methods was conducted from the health provider’s perspective. The main unit of analysis is the economic unit cost per visit for each service. Costs are converted to 2013 International dollars.

**Results:**

The mean cost per visit for the HIV/SRH services ranged from $Int 14.23 (PNC visit) to $Int 74.21 (HIV treatment visit). We found considerable variation in the unit costs per visit across settings with family planning services exhibiting the least variation ($Int 6.71-52.24) and STI treatment and HIV treatment visits exhibiting the highest variation in unit cost ranging from ($Int 5.44-281.85) and ($Int 0.83-314.95), respectively. Unit costs of visits were driven by fixed costs while variability in visit costs across facilities was explained mainly by technology used and service maturity.

**Conclusion:**

For all services, variability in unit costs and cost components suggest that potential exists to reduce costs through better use of both human and capital resources, despite the high proportion of expenditure on drugs and medical supplies. Further work is required to explore the key drivers of efficiency and interventions that may facilitate efficiency improvements.

## Introduction

The debate concerning how to best organize sexual and reproductive health (SRH) and HIV services is long standing. The integration of SRH and HIV services evolved in the 1990s in response to the rapid rise in the HIV epidemic in sub Saharan Africa, which heightened global concerns about the relative lack of services to address broader SRH problems [1]. In more recent years, a number of global policies and high-level position papers [2–7] have called for integration of HIV and SRH services, with efforts focused on integrating the prevention and treatment of sexually transmitted infections (STI) including HIV, into family planning (FP) services.

Although the benefits of integrating these services in terms of increased access to HIV services and continuity of care in these two settings have been well articulated, there remains a dearth of evidence on the costs of integrated SRH/HIV services [8–10]. In addition, little is understood of how these costs vary across facilities and settings. The few studies evaluating integration from an economic perspective to date have evaluated a small number of sites and focused only on costs of integrated counseling and testing for HIV within family planning services [11–13]. One exception is a recent study conducted in Kenya, which evaluated the average and marginal costs of family planning services integrated into HIV care and treatment services [14].

This paper therefore presents evidence on the total costs and unit costs per visit of delivering six integrated HIV and SRH services across 40 providers in Kenya and Swaziland. Accurate data on economic costs of delivering integrated HIV and SRH services are essential for a range of applications. First, empirical data on costs of these services can be used for resource requirements, programme planning and budgeting purposes. Second, these data can also be used be used by researchers interested in exploring the cost-effectiveness of HIV/SRH services and contribute to efforts aimed at improving efficiency in service provision.

## Methods

### Study setting

This study was conducted as part of the Integra initiative, a non-randomized trial aimed at evaluating the impact of different models of delivering integrated HIV and SRH services on a range of health and service outcomes [15]. The Integra Initiative sought to add to the limited evidence base on the economics of integration by estimating the average costs of integrated HIV and SRH services in Kenya and Swaziland.

**Fig 1:**
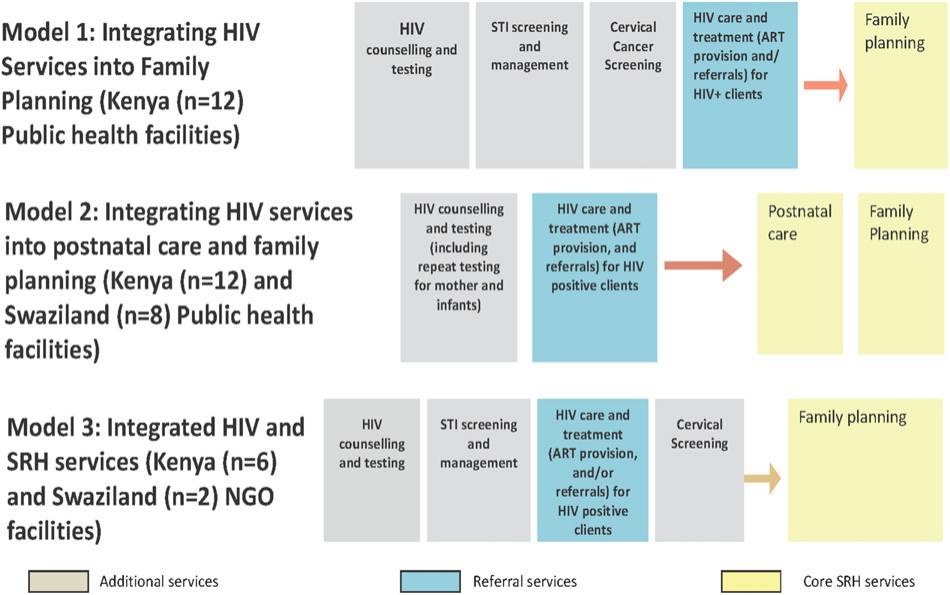
Summary of models of integration evaluated.

Broadly, the Integra initiative set out to evaluate three different models of integrating HIV and SRH service in both countries. These were: integrated FP model which promoted the integration of HIV and STI services into existing FP services; integrated post natal care (PNC) model which promoted integration of HIV and STI services into PNC/FP services; and integrated HIV/STI services provided within SRH clinics [15]. A summary of the three different models of integration is presented in Fig 1.

With national HIV prevalence rates of 26% [16] and 7.4% [17] among 15–49 year olds in Swaziland and Kenya respectively in 2007 (most recent prevalence data reported in 2013 are 5.6% [18] and 31% [19] in Kenya and Swaziland respectively) both countries considered integration of HIV and SRH services as critical to addressing the HIV crisis. Both formally adopted integration policies within their national HIV strategies [20, 21] in late 2009, aimed at providing comprehensive HIV prevention, (counseling and testing), and treatment to clients seeking maternal and child health (MCH) and FP services.

To obtain an understanding of the costs of integrated HIV and SRH services, costing of integrated HIV and SRH services was carried out in 30 health facilities in Kenya and 10 health facilities in Swaziland. The study sites were purposefully chosen from six priority regions with established programming based on previous operational research relationships with the Ministries of Health in Swaziland and Kenya [15]. Study sites varied by setting, facility type, and model of integration. In Kenya, the sites included 24 public facilities and 6 non-government organisations (NGO) affiliated SRH clinics. The public facilities were selected from two provinces (Central and Eastern) and 6 districts (Nyeri, Nyandarua, Thika, Muranga, Kitui and Makueni) and included a provincial general hospital, 5 district hospitals, 5 sub-district hospitals and 13 health centres. In Swaziland, the sites included eight public facilities and two NGO affiliated SRH clinics. The public facilities were selected from four regions (Manzini, Lubombo, Sishelweni and Hhohho) and included a public/mission district hospital, four rural health centres and three public health units (PHU). The facilities in central province of Kenya (n = 12) represented the FP model of integration, while the facilities in Swaziland and Eastern Province of Kenya (n = 20), the PNC model of integration. Although study clinics were initially chosen to represent the three different types of integration models, the study sites exhibited varying levels and extents of service integration making it difficult to make any meaningful comparisons between the three different models.

The study focused only on the MCH-FP and HIV units and integration of HIV/STI services within the MCH-FP units. HIV services evaluated in this study included treatment of STIs, counselling and testing for HIV (HCT) and HIV treatment (which included pre-antiretroviral therapy (ART), ART and treatment of opportunistic infections). SRH services included FP, PNC and cervical cancer (Ca Cx) screening routinely provided within the MCH-FP units. A detailed summary of the service descriptions is provided in Table 1.

**Table 1:** Description of SRH and HIV services included in the study.

Output	Definition
Family planning (FP)	Family planning service includes counseling on FP methods and provision of FP methods such as oral contraceptives; injectables; and long term methods such as implant, intrauterine contraceptive devices (IUCD), vasectomy and bilateral tubal ligation.
Post-natal care (PNC)	PNC services include physical check for mother and infant at 48 hrs. 7 days, 6 weeks and 6 months; Counseling on and provision of FP methods at 6 weeks; Counseling for HIV; Testing/retesting for HIV; PCR Testing for infants at 6 weeks; Counseling on danger signs for mother and new borns; Infant immunizations upto 6 months.
Cervical cancer screening (Ca Cx Screening)	Cervical cancer screening involves either the use of pap smear (a laboratory diagnostic procedure) or VIA VILLI to screen for cervical cancer.
Screening and treatment of sexually transmitted infections (STI)	STI management includes counseling, advice on sexual behavior, basic diagnosis of syndromes, partner notification, condom distribution and treatment of infections
HIV counseling and testing (HCT)	HCT includes the provision of pre-test counseling, HIV rapid testing, and post-test counseling offered to clients who either voluntarily seek HIV testing services or who receive HIV counseling and testing within the context of another health visit.
HIV treatment and care	HIV treatment and care includes a combination of psychosocial support, nutritional counseling, ARV adherence counseling, information and education on prevention strategies for PLWHA, diagnostics, provision of ARVs and treatment for opportunistic infections.

### Ethics statement

Ethics approval for the Integra study was obtained from the Ethical Committee at LSHTM (approval no. 5436), from the Population Council Review Board (protocol nos. 443 and 444), from the Kenya Medical Research Institute (approval no. KEMRI/RES/7/3/1, protocol nos SCC/113 and SCC/114) and the Swaziland Scientific and Ethics Committee (approval nos. MH/599B and MH/599C). Written informed consents were obtained for all Integra study activities.

### Data collection

A retrospective costing study was undertaken from the health provider perspective using a combination of bottom-up and step-down costing methods [22]. The bottom up costing method or ingredients based costing requires the identification and specification of each component of resource used for delivering an individual service to arrive at a total unit cost. The step-down costing method is used to allocate overhead costs or resources that serve different programs and departments. Overhead costs are allocated in a step wise fashion to all the overhead departments and then to final cost centres in this case, final HIV and SRH services [23]. Both financial and economic costs were estimated. Financial costs represent actual expenditure on goods and services while economic costs include the value of all resources used to produce output including those for which there were no financial transactions such as volunteer human resources and donated goods.

Prior to cost data collection, a periodic activity review (PAR) tool was developed to document the nature, range and methods of delivery of HIV and SRH services in each health facility. Specifically, the PAR provided an understanding of how resources are combined to produce integrated HIV and SRH services. The PAR tool included questions on facility characteristics, staffing types and levels, scope and number of services offered, descriptions of client flow, and overall description of the integration of services. The PAR tool was implemented in all SRH/HIV clinics and laboratory and pharmacies (if available onsite) from July 2010—June 2011 and April 2010—March 2011 in Kenya and Swaziland respectively.

Following the PARs in each health facility, cost and output indicators were collected retrospectively for the financial year 2010–2011. Cost data were collected for the entire facility in the health centres, public health units and SRH clinics; while in the hospitals, cost data were collected for the MCH-FP/HIV departments only.

Costs were classified into two main categories: capital and recurrent costs. Capital costs considered included buildings, equipment and training costs. All capital costs were annualized and discounted at the standard rate of 3% [22]. The facilities and departments were measured in square meters and the rooms valued using estimates on annual rental costs per square meter obtained from a rental survey of the adjacent area. Equipment and furniture inventories were developed during visits at each study site and subsequently valued using the price lists from Kenya Medical Supplies Agency and the Swaziland Ministry of Health. Most equipment was assigned a life expectancy of 3–5 years and furniture was assumed to have a longer life expectancy of 10 years.

Training related to HIV/STI and SRH service delivery was identified through interviews with service providers. Training costs were then estimated from training facilitation costs, staff per diems and transport allowances received by the service provider.

Recurrent costs included building maintenance (including utility expenses), transport costs, staff salaries drugs, diagnostics and supplies. Utility expenses were obtained from the central administration expenditure records for each facility. Personnel costs including benefits and allowances were estimated for all staff working in the MCH/PHU, HCT, HIV, pharmacy and laboratory departments, on the basis of position and salary levels. Expenditures on the recurrent costs of drugs, diagnostics and supplies were obtained from requisition notes and records within the facilities.

### Allocation of costs

Overhead and administrative costs associated with the different HIV/SRH services were allocated using the step-down costing approach. Room space was used to allocate utilities and building maintenance and staff numbers in each unit used to allocate management and administrative costs. Costs of drugs, diagnostics and supplies were allocated to each individual service based on actual resource usage. This was obtained through a combination of staff observations, staff interviews and patient records. In particular, staff time was allocated using a combination of an initial interview, followed by observations, followed by a week of timesheet reporting and then a follow-on interview to confirm allocations.

The same costing methods were used in all the 40 study sites. Two researchers collected data across sites and results were quality controlled by a third researcher. All costs were converted to standardized 2013 international dollar (Int$ 2013) using the general purchasing power parity (PPP) index [24] rather than the official currency exchange rates. The PPP index is recommended for comparing costs across countries as it adjusts for differences in relative prices between economies [25].

### Estimation of unit costs of HIV and SRH services

The main unit of this analysis is the unit cost per visit for each service, calculated by dividing the total costs for each service by the number of client visits of the respective services. Data on HIV and SRH service utilization was collected from registers and monthly reports at the facility level. In few instances where client registers were missing and service statistics were not kept, estimates of services provided were made through a review of the drug dispensing records and interviews with staff to determine average number of consultation visits made for a particular condition or need.

We present total and unit costs by country, facility type and model of integration, in order to illustrate the extent of variation in cost across a range of providers. Costs were further assessed and presented as fixed or variable costs. Variable costs include the costs of drugs, diagnostics and supplies, which vary with changes in output whereas fixed costs include capital, salaries, and other building maintenance costs that did not vary with output levels within the time period of a year.

## Results

### Total costs of HIV and SRH services

Table 2 presents the percentage breakdown of total outpatient HIV and visits and total economic costs for each of the six services by country, ownership, facility type and model type. The corresponding amounts are provided in S1 Table. One striking feature of the breakdowns is the large proportion of facility costs incurred for HIV/STI counseling, testing, and treatment services in both settings. When taken together, these services account for 23% to 74% of total HIV/SRH costs in Kenya and 30% to 96% of total costs in Swaziland.

**Table 2:** Breakdown of client visits and total costs by country, ownership, facility type and service.

2010/2011																		
Country	Kenya										Swaziland							
Ownership	Public								NGO		Public						NGO	
Facility type	Hospital(n = 1)		DH (n = 5)		SDH (n = 6)		HC (n = 12)		SRH clinics (n = 6)		Hospital (n = 1)		HC (n = 5)		PHU (n = 2)		SRH (n = 2)	
	Proportion of		Proportion of		Proportion of		Proportion of		Proportion of		Proportion of		Proportion of		Proportion of		Proportion of	
Service type	Visits	Costs	Visits	Costs	Visits	Costs	Visits	Costs	Visits	Costs	Visits	Costs	Visits	Costs	Visits	Costs	Visits	Costs
Ca Cx	7%	18.3%	2%	0.7%	1%	0.4%	2%	1.7%	9%	11.2%	1%	1.1%	0%	0.0%	0%	0.0%	2%	10.7%
FP	58%	56.9%	35%	25.4%	41%	22.0%	40%	30.3%	39%	29.0%	8%	4.1%	23%	3.1%	77%	36.6%	65%	58.0%
Post natal care	21%	2.0%	10%	3.5%	20%	3.3%	13%	5.5%	1%	0.5%	3%	2.5%	2%	0.6%	12%	14.7%	3%	1.0%
HCT	12%	17.7%	24%	11.2%	23%	7.1%	19%	6.8%	36%	14.5%	2%	1.8%	3%	0.6%	8%	4.4%	16%	7.6%
STI treatment	1%	5.1%	1%	1.4%	0%	0.3%	0%	0.2%	5%	5.6%	0%	0.0%	3%	0.4%	1%	0.8%	13%	10.2%
HIV treatment	0%	0.0%	27%	57.8%	15%	66.9%	25%	55.5%	10%	39.2%	86%	90.4%	69%	95.4%	2%	43.4%	1%	12.5%

Facility type: DH = District hospital; SDH = Sub district hospital; HC = Health centre; PHU = Public health unit

Service type: Ca Cx = Cervical Cancer Screening; FP = Family planning; PNC = Post natal care; HCT: HIV counseling and testing; STI = sexually transmitted infections

We also observed considerable variation in the relative proportions of visits and costs for the different services by health facility type. In Kenya, all the facility types provided more SRH services; compared to HIV services, with SRH services accounting for between 47% and 86% of visits and their proportion of total costs ranged from 26% to 77% across facility types. In Swaziland, SRH visits accounted for between 12% and 89% of total visits and 4% to 70% of total costs of HIV/SRH services. The public health units and SRH clinics in Swaziland provided more SRH visits compared to the hospital and health centres and in these facilities, costs for these visits together accounted for >50% of HIV and SRH service costs. SRH visits in the hospital and health centres only accounted for 4%– 8% of total HIV/SRH service costs.

### Mean unit costs per visit

Table 3 presents mean economic unit costs (including drugs and supplies) per visit for each service type by country, ownership, health facility level, location and model of integration. In general, even after adjusting for price differentials using international dollars, bivariate subgroup analysis revealed significantly higher unit costs across all service types in Swaziland compared to Kenya (p<0.001). When costs were analyzed by facility type, family planning and cervical cancer screening services were significantly different across health facility types. The mean unit cost per visit for family planning services also differed significantly by model type with the SRH clinics exhibiting higher costs (p <0.001) compared to the FP and PNC model clinics. Further, unit costs per visit for family planning, cervical cancer screening and post natal care services were significantly higher in the NGO facilities compared to the public health facilities (P<0.001). The unit costs per visit for cervical cancer screening, HCT and STI treatment were significantly lower in rural areas compared to urban areas (P<-0.05). However, beyond these we found no other consistent patterns in unit costs across study facilities with no particular facility having lower unit costs for all services compared to other facilities.

**Table 3:** Mean unit cost per service* (Int$ 2013) by country and facility type.

Service^a^	Ca Cx	FP	PNC	HCT	STI Treatment	HIV Treatment
**Overall**	$26.29[25.42]	$19.09[11.01]	$14.23[17.45]	$18.73[20.08]	$55.65[65.90]	$74.21[71.49]
**By Country (Mean [SD])**						
Kenya (n = 30)	$19.99[19.41]	$16.53[9.31]	$9.28[11.48]	$13.91[16.92	$46.36[9.22]	$46.34[32.37]
Swaziland (n = 10)	$55.31[31.72]	$26.79[12.61]	$28.05[23.82]	$34.10[22.12]	$61.15[91.39]	$133.01[95.71]
**By Ownership (Mean[SD])**						
Private (n = 8)	$41.62[31.30]	$28.93[15.31]	$15.95[17.62]	$15.58[10.11]	$34.61[21.04]	$73.14[29.37]
Public (n = 32)	$20.17[20.46]	$16.64[8.27]	$13.76[17.67]	$19.72[22.30]	$66.99[79.10]	$74.56[81.49]
**By Facility type** ^**b**^ **(Mean [SD])**						
Hospital (n = 2)	$48.10[31.68]	$20.21[14.83]	$26.39[35.96]	$29.03[18.58)	$54.40[10.87]	$30.60[43.28]
District hospital (n = 5)	$9.18[8.08]	$15.40[6.05]	$6.73[6.73]	$17.97[29.46]	$27.23[53.19]	$23.45[41.65]
SD hospital (n = 6)	$5.66[5.07]	$13.03[5.96]	$9.22[11.64]	$10.01[7.90]	$36.19[62.83]	$26.74[27.39]
Health centre (n = 17)	$12.54[20.28]	$17.86[9.49]	$13.15[19.02]	$14.73[19.98]	$11.81[24.02]	$84.62[126.58]
PHU (n = 2)	$7.05[9.97]	$16.62[1.12]	$23.81[24.39]	$24.68[38.72]	$143.65[195.45]	$109.25[105.89]
SRH Clinic (n = 8)	$41.63[31.30]	$28.92[15.31]	$15.95[17.63]	$15.58[10.11]	$30.29[23.01]	$64.37[36.82]
**By Location (Mean [SD])**						
Urban (n = 17)	$30.57[28.61]	$22.48[12.91]	$15.39[17.69]	$20.12[21.88]	$59.58[73.84]	$69.92[47.95]
Rural (n = 23)	$20.59[20.19]	$16.60[8.84]	$13.28[17.62]	$17.46[18.52]	$48.36[52.46]	$77.92[88.63]
**By Model of integration** ^**c**^ **(Mean [SD])**						
FP (n = 12)	$17.96[14.92]	$16.58[10.26]	8.84[10.13]	$15.83[20.25]	$63.02[56.30]	$16.88[17.36]
PNC (n = 20)	$22.87[26.48]	$16.68[7.12]	$16.22[20.22]	$22.67[$23.67]	$71.62[105.67]	$84.17[84.22]
SRH (n = 8)	$4.62[31.30]	$28.92[15.31]	$15.95[17.62]	$15.84[10.11]	$34.61[21.04]	$73.14[29.37]

^a^ Service type: Ca Cx = Cervical cancer screening; FP = Family planning; PNC = Post natal care; HCT: HIV counseling and testing; STI = Sexually transmitted infections

^b^ Facility type: SD Hospital = Sub District hospital; PHU = Public Health Unit

^c^ Model of integration: FP = Family Planning model (HIV/STI integrated into FP Services); PNC = Post natal care (HIV/STI services integrated into PNC services); SRH = SRH model (HIV/STI services integrated into SRH clinics)

*Mean cost per service includes drugs, diagnostics and supplies.

The mean unit costs per visit by facility type and model type for each service disguises considerable variation found between individual health facilities across the two settings, S1 Fig. The mean cost per visit across facility type varied most for HIV treatment and STI services. Family planning services had the least variation in visit costs ranging from Int$13.03 in the sub district hospital to Int$28.92 in the SRH clinic. The highest absolute difference in costs was found for STI ranging from Int$11.81 in the health centres to Int$143.65 in the public health units.

### Components of unit costs per visit

Table 4 provides breakdowns of the mean economic cost per visit for each of the six services by input category (fixed and variable costs) by country. Two noticeable features of this breakdown are: the high proportion of fixed costs across all visit types with the exception of HIV treatment visits, due to the high proportion of human resource costs. The mean fixed cost per visit for the different services accounted for between 16% to 80% (Kenya) and 26% to 92% (Swaziland) of the total mean cost per visit for the different services. Of the total fixed costs, human resources costs accounted for the largest proportion of costs ranging from 56% to 81% (Kenya) and 33% to 86% (Swaziland).

**Table 4:** Distribution of unit cost (Int$ 2013) by input type.

	Ca Cx Screening		Family planning		Post natal care		HIV C&T		STI Treatment		HIV Treatment	
Year 2010–11	Cost per visit	% of total costs	Cost per visit	% of total costs	Cost per visit	% of total costs	Cost per visit	% of total costs	Cost per visit	% of total costs	Cost per visit	% of total costs
**Kenya**												
**Fixed costs**												
Capital costs	5.20	26%	1.65	10%	1.30	14%	1.81	13%	5.56	12%	2.32	5%
Salaries cost	7.80	39%	6.94	42%	5.75	62%	6.68	48%	9.74	21%	10.19	22%
Other costs	0.80	4%	0.50	3%	0.37	4%	0.97	7%	6.03	13%	0.93	2%
***Sub total***	***13*.*79***	***69%***	***9*.*09***	***55%***	***7*.*42***	***80%***	***9*.*46***	***68%***	***21*.*33***	***46%***	***13*.*44***	***29%***
**Variable costs**												
Drugs	0.00	0%	6.45	39%	0.00	0%	0.00	0%	19.93	43%	28.27	61%
Diagnostics	6.20	31%	0.99	6%	1.86	20%	4.45	32%	5.10	11%	4.63	10%
***Sub total***	***6*.*20***	***31%***	***7*.*44***	***45%***	***1*.*86***	***20%***	***4*.*45***	***32%***	***25*.*03***	***54%***	***32*.*90***	***71%***
**Total**	**19.99**	**100%**	**16.53**	**100%**	**9.28**	**100%**	**13.91**	**100%**	**46.36**	**100%**	**46.34**	**100%**
**Swaziland**												
**Fixed costs**												
Capital costs	7.74	14%	1.61	6%	6.171	22%	6.48	19%	18.96	31%	3.99	3%
Salaries cost	19.36	35%	11.79	44%	16.83	60%	15.35	45%	12.84	21%	29.26	22%
Other costs	3.87	7%	1.07	4%	1.68	6%	2.05	6%	7.34	12%	1.33	1%
***Sub total***	***30*.*97***	***56%***	***14*.*47***	***54%***	***24*.*68***	***88%***	***23*.*87***	***70%***	***39*.*14***	***64%***	***34*.*58***	***26%***
**Variable costs**												
Drugs	-	0%	8.04	30%	2.24	8%	0.00	0%	18.35	30%	53.20	40%
Diagnostics	24.34	44%	4.29	16%	1.12	4%	10.23	30%	3.67	6%	45.22	34%
***Sub total***	***24*.*34***	***44%***	***12*.*32***	***46%***	***3*.*37***	***12%***	***10*.*23***	***30%***	***22*.*01***	***36%***	***98*.*43***	***74%***
**Total**	**55.31**	**100%**	**26.79**	**100%**	**28.05**	**100%**	**34.10**	**100%**	**61.15**	**100%**	**133.01**	**100%**

When analyzed by service type, cervical cancer screening visits had the highest proportion of fixed mean costs accounting for (69–92%) in both countries. In contrast, variable costs are the most expensive component of the mean unit cost per HIV care and treatment visit accounting for the 71% to 84% of the mean unit cost per visit.

## Discussion

This is the first study to our knowledge to provide a detailed description of the resources used to deliver integrated HIV and SRH services across different settings. Our findings show that HCT and HIV treatment costs account for a significant proportion of total health service costs in both Kenya and Swaziland. However, there is considerable variation in the unit costs, levels of fixed costs and patterns of input use in the provision of integrated HIV and SRH services between facilities suggesting considerable room to improve efficiency at both the facility and service level.

Our analysis showed that even after adjusting for differences in relative prices, there were still large disparities in unit costs between the two countries. Some variation in unit costs across the different services may be associated with site characteristics, although we found few consistent patterns across facility types. For example, in Kenya, the estimated unit costs of FP and PNC visits were consistently higher in the NGO SRH clinics compared to the other health facility types. The higher unit cost for these visits in the NGO clinics may be indicative of provision of more complex FP methods such as intrauterine contraceptive devices, implants and bilateral tubal ligations which require more equipment and staff time.

There are also service specific explanations for the differences in costs between facilities. Some of these can be explained by the technology used. For example the wide range in unit costs per family planning and cervical cancer screening visits result from the wide variation in methods provided within the facilities. The underlying data confirm that facilities providing more long term FP methods and pap smears for cervical cancer screening as opposed to visual inspection had higher unit cost per visit.

Besides the method mix, most of the variability in costs stemmed from the variability in the level of fixed rather than variable unit costs. The high proportion of fixed costs as a proportion of total unit costs, suggests that there is a mismatch between planning of fixed resources and the demand for services. It should also be noted that, despite the fact that HIV care and treatment has the highest proportional mean unit variable cost per visit, it has one of the highest absolute level of fixed unit cost per visit. From an HIV programme perspective drugs and variables costs may be of most concern to planners. However, given the overall percentage of HIV care and treatment related visits particularly in the higher level facilities in Swaziland costed, HIV care and treatment related fixed costs may be key to the planning of fixed resources at the facility level.

Another explanation for service specific variation may also be service maturity [26]. This may explain why well-established services, such as FP, exhibited less variation than new services such as HIV and STI treatment that may not yet have achieved high levels of visits. It also may in part explain why integration has not been successful in ironing out cost variation as when new services are first added, service volumes may be low in a facility and hence result in higher unit costs since fixed costs are spread across few units of output. This may resolve itself in time, but it may still be necessary to examine more closely the assumptions made, if any, about the implicit level of demand for integrated services.

Mean unit costs estimated in our study for HCT and HIV treatment visits in Kenya and Swaziland did not differ greatly from costs estimated in previous studies within sub Saharan Africa [27–29] from smaller and less integrated settings. However, care should be taken in comparing costs across studies, as costs vary considerably over time, due to changes in input costs. In relation to cost structures, the results of this study are also consistent with other studies, which found high fixed costs as a proportion of total costs for most HIV and SRH services [28–31]. In a South African study of 4 sites, Rosen et al, [30] estimated that fixed costs accounted for 25% to 46% of outpatient HIV treatment costs. Similarly, in a Zambian study of HIV services in 12 health centres and hospitals, Bratt et al, [29] estimated that fixed costs accounted for 13% -62% of total costs across the different services.

Some limitations of this analysis should be noted. While the study incorporates high quality micro-costing methods rarely used in low and middle income countries, routine monitoring data was used to estimate the unit costs. Although this was partially validated through comparison with other study instruments from the broader study, it is likely that the use of routine services will bias our results. There may be an incentive for example to over-report visits that may result in lower unit costs; or alternatively reporting may be incomplete, that would result in an over-estimation of unit costs. This reporting may also vary by service. A particular concern is the reporting of STI visits, which were not uniformly recorded across sites resulting in higher unit costs estimated for these services.

Secondly, this analysis excludes the above service delivery costs or costs incurred at the administrative level outside the point of service delivery which may comprise an important component of costs, particularly fixed costs [32]. Such costs would provide valuable insights into approaches for optimizing resource management and health system costs. Given that above service level activities can contribute substantially to overall costs of services and are likely to provide opportunities for sharing of fixed costs in the process of integration (e.g. integrated management of information systems), future cost studies should focus specifically on these costs.

Notwithstanding these limitations, the findings of this study yield important policy and practice implications regarding the optimization of health resources to improve efficiency at the health facility level. First, this study indicates that the current level of efficiency of integrated HIV and SRH services can still be improved. Second, the study findings suggest that on its own, integration does not resolve the issue of cost variation between services, although more work is required to isolate the specific impact of integration efforts. However, given that fixed costs account for a significant proportion of unit costs particularly for SRH services, and vary considerably between sites, integrated delivery of HIV and SRH services still offers the potential for better use of resources. It also suggests that in some settings, the fixed capacity exists to absorb this extra demand, but it is also clear that even when a policy of integration has been adopted this is not always achieved in practice. Further guidance is therefore required for facility managers on staffing services, not solely from a clinical perspective, but also taking into account the staff workloads and local demand for different HIV/SRH services.

Finally our findings highlight the complexity of the factors that may influence costs. Many of these issues are hard to address at the national level, yet managers at the facility and district level rarely have access to data on the underlying costs of the inputs and outputs they provide. Despite the fact significant investments have been made in decentralizing health systems, it is still difficult to find the necessary data to conduct and interpret even the simplest costing at the local level; and without this, it is unreasonable to expect that managers integrating services are able to move towards the lowest cost model. Policy makers and planners should therefore focus on strengthening simple information systems that match cost with financial information which would help managers identify local solutions that fully reflect the range of factors driving inefficiencies.

## Conclusion

This study provides the most complete evidence to date on the unit costs of HIV and SRH services, in a variety of facilities, across a medium and high HIV prevalence setting including a unique description of the cost breakdowns for each visit type. The considerable variation in unit costs of integrated HIV and SRH services found suggests a potential to improve efficiency. Given the large proportion of fixed costs for most of the services, if potential efficiency gains are to be realized, better use of existing human resources at the facility level should be advocated alongside integration policies and generation of demand for services. Finally, while this study has provided an important characterization of the costs of different HIV and SRH services in multiple sites, further research and analysis of these data is required to examine the determinants of costs, including whether the extent of integration has an impact on costs.

## Supporting Information

S1 Fig Variation in mean cost per visit* by service type. * Mean cost per visit includes drugs, diagnostics and supplies.
(TIF)

S1 Table Breakdown of HIV and SRH Service average visits and average costs (Int $ 2013) by country and health facility level. (PDF)

## References

[1] Lush L. Service integration: an overview of policy developement. Issues in Perspective. 2002;28(2):71–6.

[2] UNGASS. Declaration of commitment on HIV/AIDS. New York: UNITED NATIONS GENERAL ASSEMBLY SPECIAL SESSION ON HIV/AIDS 25–27 6 2001, 2001.

[3] UNFPA. The Glion Call to Action on Family Planning and HIV/AIDS in Women and Children, 3–5 5 2004 New York, NY: United Nations Population Fund, 2004.

[4] UNFPA. The New York Call to Commitment: Linking HIV/AIDS and Sexual Reproductive Health. 2004.

[5] Gleneagles G. The Gleneagles Comminique on Africa. 2005.

[6] UNGASS. Resolution 60/262. Political Declaration on HIV/AIDS New York, NY: 2006.

[7] UNGASS. Resolution 65/277.Political Declaration on HIV and AIDS: Intensifying Our Efforts to Eliminate HIV and AIDS. 2011.

[8] Sweeney S, Obure CD, Maier CB, Greener R, Dehne K, Vassall A. Costs and efficiency of integrating HIV/AIDS services with other health services: a systematic review of evidence and experience. Sex Transm Infect. 2011. 10.1136/sextrans-2011-050199 22158934

[9] Askew I, Berer M. The Contribution of Sexual and Reproductive Health Services to the Fight against HIV/AIDS: A Review. Reproductive Health Matters. 2003;11(22):51–73. 14708398 10.1016/s0968-8080(03)22101-7

[10] Church K, Mayhew SH. Integration of STI and HIV Prevention, Care, and Treatment into Family Planning Services: A Review of the Literature. Studies in Family Planning. 2009;40(3):171–86. 19852408 10.1111/j.1728-4465.2009.00201.x

[11] Das R, Biswas K, Panda P, Khan ME, Homan R. Strengthening Financial Sustainability through Integration of Voluntary Counseling and Testing Services with Other Reproductive Health Services. Washington DC: Population Council, 2007.

[12] Liambila W, Askew I, Ayisi R, Gathitu M, Mwangi J, Homan R, et al Feasibility, acceptability, effect and cost of integrating counseling and testing for HIV within family planning services in Kenya. Washington DC: Population Council, 2008.

[13] Homan R, Mullick S, Nduna M, Khoza D. Cost of introducing two different models of integrating VCT for HIV within family planning clinics in South Africa Linking Reproductive Health, Family Planning, and HIV/AIDS in Africa, 9–10 10; Addis Ababa 2006.

[14] Shade SB, Kevany S, Onono M, Ochieng G, Steinfeld RL, Grossman D, et al Cost, cost-efficiency and cost-effectiveness of integrated family planning and HIV services. AIDS. 2013;27 Supplement(1):S87–S92. 24088688 10.1097/QAD.0000000000000038

[15] Warren C, Mayhew S, Vassall A, Kimani JK, Church K, Obure CD, et al Study protocol for the Integra Initiative to assess the benefits and costs of integrating sexual and reproductive health and HIV services in Kenya and Swaziland. BMC Public Health. 2012;12(1):973. 23148456 10.1186/1471-2458-12-973 PMC3529107

[16] Central Statistics Office. Swaziland Demographic and Health Survey 2006–07. In: Office CS, editor. Mbabane, Swaziland: Central Statistical Office 2007 doi: 10.1371/journal.pone.0117219

[17] National AIDS and STI Control Programme MoH, Kenya. 2007 Kenya AIDS Indicator Survey: Final Report. Nairobi, Kenya: NASCOP, 2009.

[18] National AIDS and STI Control Programme MoH, Kenya. Kenya AIDS Indicator Survey 2012: Preliminary Report. Nairobi, Kenya. 2013 September 2013. Report No.

[19] Government of Swaziland. Swaziland HIV incidence measurement survey: First findings report 2012. 2013.

[20] Government of Kenya. National Reproductive Health and HIV and AIDS Integration Strategy. Nairobi, Kenya: Government of Kenya; 2009.

[21] Government of Swaziland. National Strategic Framework (NSF) on HIV and AIDS for 2009–2014. Mbabane, Swaziland: 2009.

[22] Drummond M, Sculpher M, Torrance G, O'Brien B, Stoddart, Greg L. Methods for the economic evaluation of healthcare programmes. Third ed Oxford: Oxford University Press; 2005.

[23] Conteh L, Walker D. Cost and unit cost calculations using step-down accounting. Health Policy Plan. 2004;19(2):127–35. 14982891 10.1093/heapol/czh015

[24] World Bank. PPP conversion factor, GDP (LCU per international $). Available from: http://data.worldbank.org/indicator/PA.NUS.PPP. (Accessed 30 December 2014)

[25] Kanavos P, Mossialos E. International Comparisons of Health Care Expenditures: What We Know and What We Do not Know. Journal of Health Services Research & Policy. 1999;4(2):122–6. 10387404 10.1177/135581969900400211

[26] Menzies NA, Berruti AA, Blandford JM. The Determinants of HIV Treatment Costs in Resource Limited Settings. PLoS ONE. 2012;7(11):e48726 doi: 10.1371/journal.pone.0048726 23144946 10.1371/journal.pone.0048726 PMC3492412

[27] Cleary S, McIntyre D, Boulle A. The cost-effectiveness of Antiretroviral Treatment in Khayelitsha, South Africa—a primary data analysis. Cost Effectiveness and Resource Allocation. 2006;4(1):20. 17147833 10.1186/1478-7547-4-20 PMC1770938

[28] Bikilla A, Jerene D, Robberstad B, Lindtjorn B. Cost estimates of HIV care and treatment with and without anti-retroviral therapy at Arba Minch Hospital in southern Ethiopia. Cost Effectiveness and Resource Allocation. 2009;7(1):6. 19364399 10.1186/1478-7547-7-6 PMC2672061

[29] Bratt JH, Torpey K, Kabaso M, Gondwe Y. Costs of HIV/AIDS outpatient services delivered through Zambian public health facilities. Tropical Medicine & International Health. 2011;16(1):110–8. 20958891 10.1111/j.1365-3156.2010.02640.x

[30] Rosen S, Long L, Sanne I. The outcomes and outpatient costs of different models of antiretroviral treatment delivery in South Africa. Tropical Medicine & International Health. 2008;13(8):1005–15. 18631314 10.1111/j.1365-3156.2008.02114.x

[31] Martinson N, Mohapi L, Bakos D, Gray GE, McIntyre JA, Holmes CB. Costs of Providing Care for HIV-Infected Adults in an Urban HIV Clinic in Soweto, South Africa. JAIDS Journal of Acquired Immune Deficiency Syndromes. 2009;50(3). 10.1097/QAI.0b013e3181958546 PMC3080749 19194308

[32] Johns B, Baltussen R, Hutubessy R. Programme costs in the economic evaluation of health interventions. Cost Effectiveness and Resource Allocation. 2003;1(1):1 12773220 10.1186/1478-7547-1-1 PMC156020

