# Supplementary figures and images for: The Costs of Delivering Integrated HIV and Sexual Reproductive Health Services in Limited Resource Settings
Source: PLoS One. 2015 May 1;10(5):e0124476. doi: 10.1371/journal.pone.0124476 (PMC4416893; doi:10.1371/journal.pone.0124476)

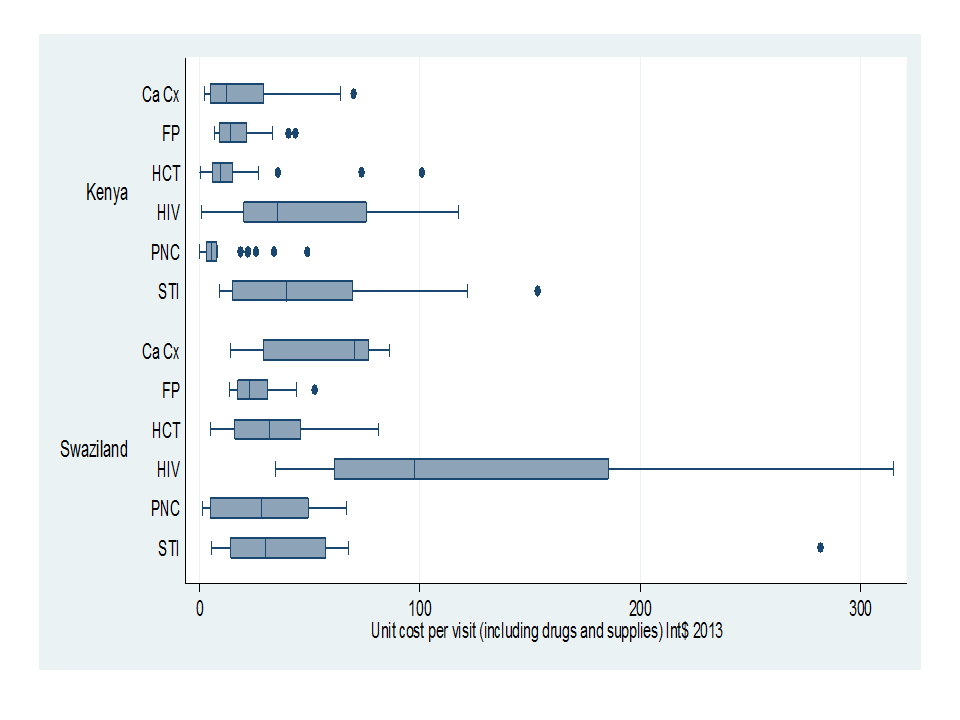

Supplement: S1 Fig — * Mean cost per visit includes drugs, diagnostics and supplies. (TIF) [file pone.0124476.s001.tif]
