# Supplementary material for: The Costs of Delivering Integrated HIV and Sexual Reproductive Health Services in Limited Resource Settings
Source: PLoS One. 2015 May 1;10(5):e0124476. doi: 10.1371/journal.pone.0124476 (PMC4416893; doi:10.1371/journal.pone.0124476)
Supplement: S1 Table — (PDF) [file pone.0124476.s002.pdf]

S1 Table: Breakdown of HIV and SRH Service average visits and average costs (Int $ 2013) by country and health facility level

Country	Kenya									
Facility type	Hospital (n=1)		District Hospital (n=5)		Sub District Hospital (n=6)		Health Centre (n=12)		SRH Clinic (n=6)	
	Visits	Average Costs	Visits	Average Costs	Visits	Average Costs	Visits	Average Costs	Visits	Average Costs
Ca Cx	806	$20,120	265	$2,059.87	71	$475	54	$1,196	852	$24,714
FP	6636	$62,662	5438	$78,675	2386	$24,069	1448	$21,047	3500	$64,097
PNC	2436	$2,256	1541	$10,943	1166	$3632	483	$3,789	73	$1,006
HCT	1421	$19,497	3739	$34,723	1323	$7,768	679	$4,712	3298	$32,175
STI treatment	93	$5,608	224	$4,383	5	$322.15	12	$146.91	465	$12,477
HIV treatment*	-	0	4164	$179,443	867	$73,383	905	$38,496	867	$86,747

Country	Swaziland							
Facility type	Hospital (n=1)		Health Centre (n=5)		Public Health Unit (n=2)		SRH (n=2)	
	Visits	Average Costs	Visits	Average Costs	Visits	Average Costs	Visits	Average Costs
Ca Cx	756	$42,799	13	$163	10	127.69	302	$58,814
FP	6379	$157,197	4645	$67,395	16314	$218,834	11042	$319,300
PNC	2319	$96,498	455	$13,102	2524	$87,849	463	$5,552
HCT	1947	$69,547	591	$12,874	1600	$26,222	2698	$41,628
STI treatment	15	$562	669	$8,258	214	$4,742	2284	$56,392
HIV treatment	70605	$3,469,352	14203	$2,102,757	457	$259,411	231	$68967

*No data on HIV care and treatment visits were collected at the hospital
